# From Crop Residue to Corrugated Core Sandwich Panels as a Building Material

**DOI:** 10.3390/ma18010031

**Published:** 2024-12-25

**Authors:** Aadarsha Lamichhane, Arun Kuttoor Vasudevan, Mostafa Mohammadabadi, Kevin Ragon, Jason Street, Roy Daniel Seale

**Affiliations:** Department of Sustainable Bioproducts, Mississippi State University, P.O. Box 9820, Starkville, MS 39762, USA; al2409@msstate.edu (A.L.); ak2089@msstate.edu (A.K.V.); kwr2@msstate.edu (K.R.); jts118@msstate.edu (J.S.); rds9@msstate.edu (R.D.S.)

**Keywords:** crop residues, small-diameter trees, biobased materials, rice husks, wheat straws, wood strands, sheathing, flooring, experimental testing, bending performance

## Abstract

This study explores the potential of using underutilized materials from agricultural and forestry systems, such as rice husk, wheat straw, and wood strands, in developing corrugated core sandwich panels as a structural building material. By leveraging the unique properties of these biobased materials within a corrugated geometry, the research presents a novel approach to enhancing the structural performance of such underutilized biobased materials. These biobased materials were used in different lengths to consider the manufacturing feasibility of corrugated panels and the effect of fiber length on their structural performance. The average lengths for wood strands and wheat straws were 12–15 cm and 3–7.5 cm, respectively, while rice husks were like particles, about 7 mm long. Due to the high silica content in rice husk and wheat straw, which negatively impacts the bonding performance, polymeric diphenylmethane diisocyanate (pMDI), an effective adhesive for such materials, was used for the fabrication of corrugated panels. Wood strands and phenol formaldehyde (PF) adhesive were used to fabricate flat outer layers. Flat panels were bonded to both sides of the corrugated panels using a polyurethane adhesive to develop corrugated core sandwich panels. Four-point bending tests were conducted to evaluate the panel’s bending stiffness, load-carrying capacity, and failure modes. Results demonstrated that sandwich panels with wood strand corrugated cores exhibited the highest bending stiffness and load-bearing capacity, while those with wheat straw corrugated cores performed similarly. Rice husk corrugated core sandwich panels showed the lowest mechanical performance compared to other sandwich panels. Considering the applications of these sandwich panels as floor, wall, and roof sheathing, all these panels exhibited superior bending performance compared to 11.2 mm- and 17.42 mm-thick commercial OSB (oriented strand board) panels, which are commonly used as building materials. These sandwich structures supported a longer span than commercial OSB panels while satisfying the deflection limit of L/360. The findings suggest the transformative potential of converting renewable yet underutilized materials into an engineered concept, corrugated geometry, leading to the development of high-performance, carbon-negative building materials suitable for flooring and roof applications.

## 1. Introduction

Agriculture is the world’s largest industry, employing over one billion people and generating more than USD 1.3 trillion worth of food annually [1]. Covering about 38% of the world’s land area, agricultural production provides nearly 30% of the world’s primary products, crucial for sustaining human life [2]. Among these crops, rice and wheat are vital to global food security, with rice being the staple food for more than half of the world’s population [3]. Approximately 480 million metric tons of milled rice are produced annually, with the demand expected to reach 800 million tons by 2025, according to estimates by the International Rice Research Institute (IRRI) [4,5]. Wheat, another fundamental crop, is grown on a large scale to meet global demand, with current production at 642 million tons and projected to reach 840 million tons by 2050 [6]. This significant rice and wheat production leads to vast quantities of agricultural waste in the form of straw and husk, which pose environmental challenges due to improper disposal.

Rice husk (RH) is a byproduct of rice milling, which results in about 150 million tons of husk annually [7]. RH is primarily used as fuel in rice mills for steam generation due to its high energy value, but this also leads to the production of rice husk ash (RHA), a waste product that is often improperly disposed of [8,9]. The improper disposal of RHA contributes to environmental pollution, as burning RH releases harmful black carbon and silica particles that exacerbate air quality issues [10]. Similarly, wheat and rice straws, which are left over after harvesting, are often burned in open fields, releasing significant amounts of carbon monoxide, carbon dioxide, and nitrogen oxide, all of which contribute to air pollution [11,12]. These environmental issues highlight the need for sustainable solutions that repurpose agricultural waste and reduce its harmful impacts.

One potential solution for reducing the environmental impact of agricultural waste is exploring such materials in developing long-lived products to sequester the stored carbon. Unlike short-lived products, such as fuel or animal bedding, which release stored carbon back into the atmosphere, structural products can help lock in carbon for extended periods, contributing to a more sustainable solution. Recent research has focused on repurposing agricultural residues, such as rice husk and wheat straw, for sustainable building materials, like particle board and fiberboard. Pradhan et al. evaluated the production of rice husk composite boards using various adhesives and processing methods. Some researchers have explored the fabrication of particle boards with rice husk [13,14,15,16], and similar studies have investigated the use of wheat straw in particle board [17,18,19,20]. Additionally, there have been advancements in flooring materials made from these residues. Ayitea et al. studied the development of hollow rice husk blocks specifically designed for flooring applications [21].

Small-diameter trees, often underutilized in the forestry system, are processed into flakes to manufacture oriented strand board (OSB). This product is widely used as sheathing for floors, walls, and roofs in the building industry. Since its introduction in the 1980s, OSB has gradually replaced plywood in the market, gaining widespread popularity for its strength, durability, and cost-effectiveness. Sheathing panels are typically installed over studs, joists, or rafters to distribute the load applied to walls, flooring, and roofing, transferring to the structural framing and foundation. Incorporating agricultural residues, such as rice husk and wheat straw, to develop high-performance sheathing panels offers a promising solution to countries with limited forest resources. This approach promotes more efficient use of our resources and helps to reduce the construction industry’s environmental footprint.

Researchers have used engineering concepts like corrugated geometries to develop high-performance products from underutilized biobased materials. This configuration not only makes structures lightweight but also reduces material consumption and enhances both acoustic and thermal insulation due to its hollow form [22,23]. Incorporating corrugated geometry with biobased materials has gained considerable attention in construction applications, including floor slabs, wall panels, and beams. Gaudelas et al. investigated the physical properties of corrugated panels made from wood veneer and kraft paper [24]. Corrugated cardboard has also been utilized as a core material in sandwich panels designed for building applications [25,26]. Additionally, several studies have focused on the structural performance of concept of sandwich panels with corrugated cores made from oriented strands, underscoring the potential of these materials for use in construction [27,28,29].

This study aims to investigate the potential for producing corrugated core sandwich panels using agricultural waste products, such as rice husk and wheat straw, as well as wood strands from small-diameter trees. By repurposing these abundant waste materials, this research focuses on the manufacturing feasibility and comparison of their structural properties with the commercially available OSB. Since bending is a common load applied on building materials, the corrugated core sandwich panels will be submitted to a four-point bending test to evaluate their bending performance. This approach can potentially transfer renewable and underutilized crop residues into high-performance structural products suitable for the building industry.

## 2. Materials and Methods

### 2.1. Corrugated and Flat Panels

**Figure 1:**
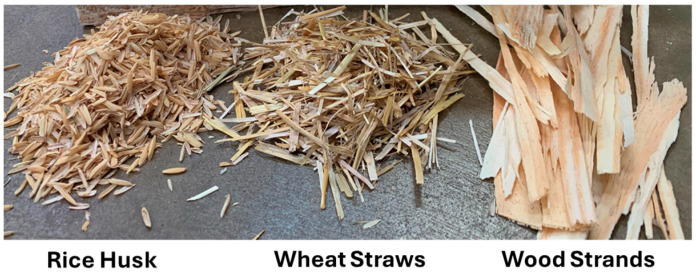
Rice husk, wheat straws, and wood strands with different fiber lengths.

Underutilized raw materials from both agricultural and forestry systems were used to fabricate corrugated panels. To investigate the impact of fiber length on both the manufacturing process and the structural performance of corrugated panels, biobased materials with varying fiber lengths were selected. Rice husks were like particles with an average length of 7 mm; whereas, wheat straws had an average length of 3–7.5 cm. Commercial southern yellow pine strands produced by processing small-diameter trees had an average length of 12–15 cm and were supplied by West Fraser, Guntown, MS, USA. A comparison of these biobased materials with different fiber lengths is presented in Figure 1. Rice husks and wheat straws are among biobased materials with high silica content [30,31]. Silica negatively impacts the bonding performance, and the extent of this impact depends on the type of adhesive used. Polymeric diphenylmethane diisocyanate (pMDI) (Rubinate 1840 supplied by Huntsman, The Woodlands, TX, USA) adhesive, which is formaldehyde-free, which results in good bonding performance for high silica content materials [32], was used to fabricate corrugated panels. The moisture content of rice husks, wheat straws, and wood strands was about 10%, 11%, and 9%, respectively. Since pMDI adhesive provides better bonding when biobased raw materials have a moisture content of around 10%, no additional drying was required. The pMDI adhesive was sprayed onto the raw materials inside a drum blender, targeting a resin content of 5% based on the oven-dry weight of the materials. Rice husks and wheat straws coated with pMDI resin were evenly placed inside a forming box to make a biobased mat, known as preform, with an even thickness. Since wood strands were long enough, they were manually aligned parallel to each other using an orienter inside the forming box to create a wood strand mat. The orientation of the wood strands allows for achieving higher mechanical properties in a specific direction. In this study, wood strands were aligned parallel with the length of corrugated panels, which is perpendicular to the corrugation axis. It is worth noting that, due to the short length of rice husks, there was no interlocking effect between them. As a result, the mat was loose and difficult to handle, presenting a significant practical challenge for scaling up the production of rice husk corrugated panels.

**Figure 2:**
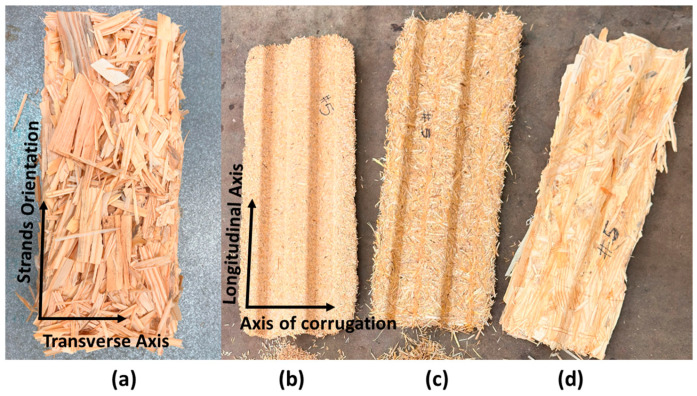
(**a**) Strand orientation for wood panels (**b**) rice husk, (**c**) wheat straw, and (**d**) wood strand corrugated panels.

The preform was placed between the halves of an aluminum matched-die mold to be formed into a corrugated geometry. The mat for each biobased material was hot-pressed at 145 °C for 5 min to a target density of 720 kg/m^3^. The orientation of wood strands as well as uniaxial corrugated panels with a wall thickness of 6.35 mm and a total depth of 25.4 mm are shown in Figure 2.

### 2.2. Sandwich Panels

Flat panels were fabricated to serve as the outer layers of the sandwich panels. To create a structural product capable of bearing the high normal stresses generated in the outer layers under bending loads, wood strands with long fiber lengths were utilized to fabricate these outer layers. Since silica is not a concern for wood strands, phenol-formaldehyde (PF) resin supplied by Hexion, Diboll, TX, USA, was used to manufacture the flat panels. The wood strands were dried in an oven to a moisture content of 3–5%, and then, PF resin was sprayed onto the strands, targeting a content of 5% based on their oven-dry weight. Using the orienter, the wood strands were manually aligned parallel to each other to form the mat. The mat was then pressed at 160 °C for 5 min, achieving a target density of 720 kg/m^3^ and a thickness of 6.35 mm.

**Figure 3:**
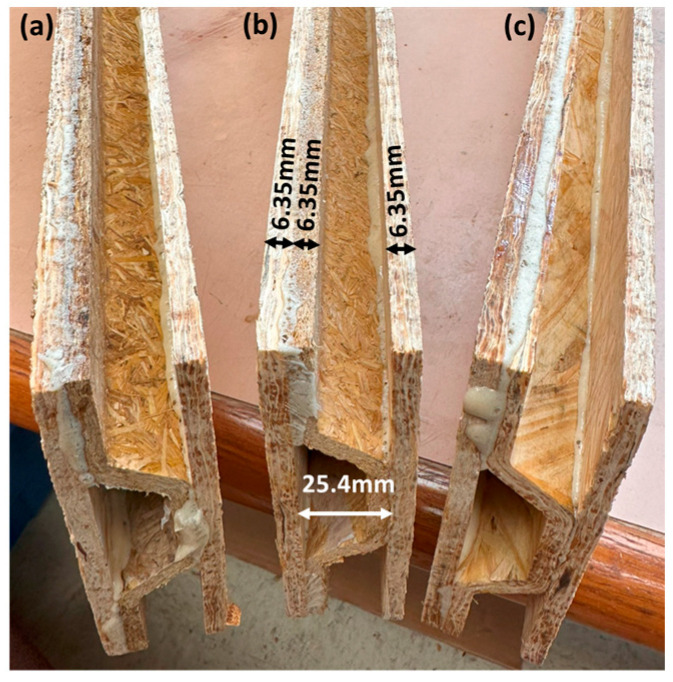
Sandwich panels fabricated from (**a**) wheat straw, (**b**) rice husk, and (**c**) wood strand corrugated cores and flat wood strand outer layers.

**Figure 4:**
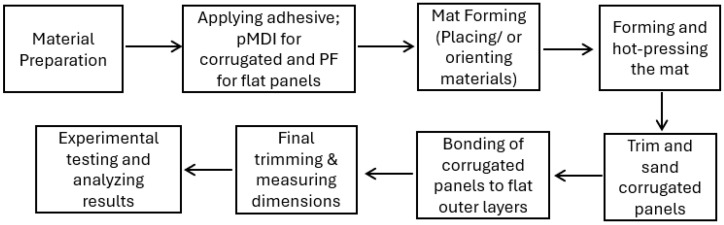
Flowchart summarizing the fabrication and testing processes of corrugated panels.

The corrugated and flat panels were sanded to improve bonding performance by enhancing surface smoothness and removing contaminants, such as the release agent applied to the mold and press surfaces. The corrugated cores were bonded to flat wood strand-based panels on both sides using a commercially available polyurethane adhesive, known as original Gorilla Glue, Cincinnati, OH, USA, for the sandwich panel fabrication, which is a common practice in OSB production. The entire assembly was cold-pressed and left under pressure for 1.5 h to allow the resin to set fully, ensuring strong bonding. The sandwich panels were then trimmed, as shown in Figure 3. The flowchart summarizing the fabrication and testing process of the corrugated panels is shown in Figure 4.

### 2.3. Experimental Testing

Considering their applications as roof, floor, or wall sheathing, the design capacity of these sandwich panels is mainly controlled by their bending performance under live, dead, snow, and wind loads. Following ASTM D7249 standard guidelines [33], these sandwich panels were submitted to a four-point bending test. Given that 41 cm (16 inches) and 61 cm (24 inches) are very common spans for sheathing panels, these dimensions were selected to evaluate the bending performance of the sandwich panels. The average dimensions of the sandwich panels are given in Table 1. Five specimens of each panel type were tested. For longer specimens, the loading span was one-third of the span length, L/3, known as the third-point bending test. Due to the limitation on our loading head, the load span for short specimens was slightly more than one-third of the span length, setting at 152.4 mm (6 inches), while the distance between the end supports and loading points was 127 mm (5 inches). Unlike other types of sheathing, floor panels are subjected to greater bending loads, both dead and live loads, and thus require a stricter deflection limit. Considering the raw materials and intended applications, OSB was identified as a closely related product for comparison. Therefore, commercially available and APA-rated floor sheathing, oriented strand board (OSB), with two different thicknesses and span lengths, was purchased and tested for comparison. Boards with a thickness of 11.2 mm were rated for a span length of 41 cm (16 inches), while those with a thickness of 17.42 mm were rated for a span length of 61 cm (24 inches). Observations were recorded for the maximum applied load, failure modes, and the deflection of the sandwich panels for further analysis. Additionally, bending stiffness (EI) was determined based on the given Equation (1).

(1) $$EI=\frac{Pa}{48∆}(3L^{2}-4a^{2})$$

The primary loads used for the design of building materials are dead load and live load in the form of distributed load. Since the maximum bending moment is an important factor dictating the load-carrying capacity, it was used to convert the bending load obtained from the 4-point bending test into an equivalent distributed load. Assuming the equal maximum moment of distributed and point loads, the equivalent maximum distributed load was determined using Equation (2).

$$\mathrm{Maximum}\;\mathrm{bending}\;\mathrm{moment}\;\mathrm{for}\;\text{four-point}\;\mathrm{bending}\;\mathrm{load},\;M_{max,4-point}\;=\;\frac{Pa}{2}$$

$$\mathrm{Maximum}\;\mathrm{bending}\;\mathrm{moment}\;\mathrm{for}\;\mathrm{distributed}\;\mathrm{bending}\;\mathrm{load},\;M_{max,distributed}\;=\;\frac{{wL}^{2}}{8}$$

(2) $$\mathrm{Equivalent}\;\mathrm{distributed}\;\mathrm{load},\;M_{max,4-point}=M_{max,distributed}\;\to \;w_{eq}=\frac{4Pa}{L^{2}}$$

Using the bending stiffness (EI) calculated using Equation (1), the span length of L for defection limits of ∆ = L/240 or L/360 for specimens under a uniformly distributed bending load of w was calculated using Equation (3).

(3) $$∆=\frac{5{wL}^{4}}{384EI}$$

where E = modulus of elasticity;

P = concentrated applied load on the beam;

I = the second moment of area (moment of inertia) of the beam’s cross-section;

∆ = deflection of the beam at the mid-span;

L = span length;

a = distance from the load to the nearest support;

w_eq_ = equivalent maximum distributed load;

w = uniformly distributed load.

## 3. Results and Discussion

To fabricate corrugated panels, the biobased mat was hot-pressed between the mold’s halves to the target density of 720 kg/m^3^ and wall thickness of 6.35 mm. However, when biobased products are released from the compression force of the hot press, their wall thickness tends to increase, a phenomenon known as springback [34]. This change in thickness due to the springback also impacts the target density of the products [35]. The increase in density can be attributed to the amount of furnish used in manufacturing the corrugated panels. The volume and surface area of the corrugated panels were used to calculate the required raw material. Due to their larger surface area compared to flat panels, corrugated panels require more material for fabrication, leading to a higher density. The actual density and wall thickness of these biobased corrugated panels are presented in Table 2.

Elastic compression stresses and the resulting deformation are the primary factors behind springback, as they cause the biobased materials to revert to their original shapes. In addition to thickness changes, the length of biobased materials, which influences the interlocking effect, is another key factor in controlling the density. When biobased materials are well interlocked, the mat becomes easier to handle, and during hot-pressing, the materials do not spread out, maintaining the target density.

Compared to wheat straw and wood strand panels, rice husk panels demonstrated the lowest density for two reasons: (a) there is no interlocking mechanism between rice husks, causing them to spread out during hot-pressing, resulting in panels with greater width and length than the target dimensions, and (b) due to the oval geometry of rice husks, greater linear compression deformation was stored in them, leading to relatively higher springback. Therefore, the final dimensions of the rice husk panel—length, width, and wall thickness—exceeded the target dimensions, leading to an increased volume and reduced density. Wheat straw corrugated panels experienced less springback compared to wood strand panels, as the raw wheat straws, with an average thickness of 0.23, were thinner than wood strands, which measured 0.98 mm. The thicker materials store more linear compression deformation, leading to a greater springback effect [36]. Despite the greater springback in wood strand panels, leading to a larger volume and smaller density, they exhibited a greater density compared to wheat straw panels, which contradicts our initial expectation. This can be attributed to the length of the raw materials and the interlocking effect. Since wood strands were longer than wheat straws, they interlocked more effectively and did not spread out during the hot-pressing process. As a result, the final length and width of wheat straw panels were larger than those of wood strands, leading to a greater final volume and, consequently, a lower density.

**Figure 5:**
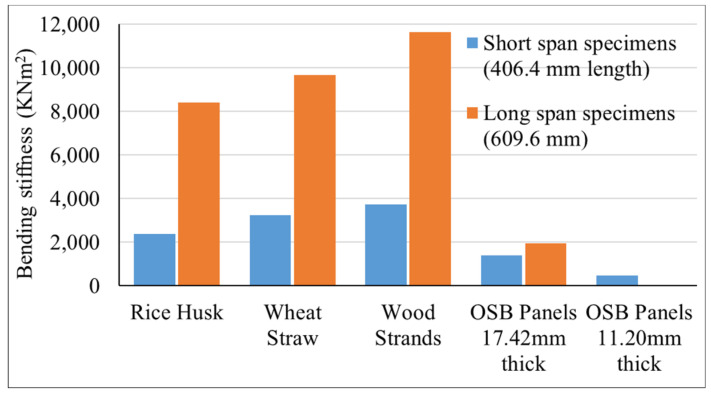
Bending stiffness for short and long span panels made up of rice husk, wheat straw, wood strands, and OSB panels of different thicknesses.

The results from the 4-point bending tests for corrugated core sandwich panels and flat OSB panels of varying thicknesses across short and long spans were evaluated in terms of bending stiffness (EI), maximum equivalent distributed load, and failure modes. Each material exhibited distinct mechanical behavior, influenced by its composition and particle orientation. The bending stiffness for all short- and long-span panels is presented in Figure 5. Overall, sandwich panels incorporating corrugated cores showed significantly greater EI values compared to flat panels. Sandwich panels with wood strand corrugated cores exhibited significantly elevated bending stiffnesses—approximately 168% greater for short-span specimens and 496% higher for long-span specimens—compared to the 17.42 mm (0.75-inch)-thick flat OSB panel. The enhanced stiffness of the sandwich panels can be attributed to their geometric structure, which increases the moment of inertia and improves resistance to bending. The corrugated geometry provided superior bending performance compared to flat panels. Additionally, longer-span specimens demonstrated higher EI values for the same material and geometry than shorter ones. This can be attributed to the increased dominance of bending performance in longer spans or the increased dominance of shear deformation in short specimens. As the span length-to-depth ratio increases, the effect of shear deformation decreases, resulting in better bending stiffness for the same specimen and material. Beams with high length-to-depth ratios fall under the Euler–Bernoulli beam theory, which neglects the shear effect. In contrast, those with smaller length-to-depth ratios are evaluated using the Timoshenko beam theory, which considers the shear effect.

Comparing the sandwich panels, those with rice husk and wheat straw corrugated cores showed lower bending stiffness than wood strand corrugated cores. The bending stiffness of rice husk and wheat straw panels was 36% and 13% lower than that of wood strands for shorter spans and 28% and 17% lower for longer spans, respectively. The difference in bending stiffness among sandwich panels is attributed to the variation in fiber length of biobased materials used in the fabrication of corrugated cores. Wood strands, averaging 12–15 cm in length, produced corrugated sandwich panels with the highest bending stiffness, while rice husks, with an average length of only 7 mm, resulted in the lowest bending stiffness. Wheat straw, measuring 3–7.5 cm, fell in between the performance of wood strands and rice husks. Longer fiber interlocked better and increased the mechanical performance.

**Figure 6:**
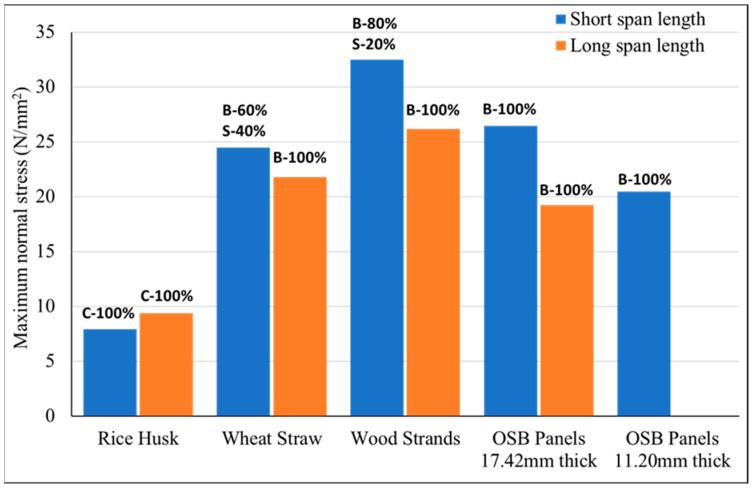
Maximum normal stress for agricultural wastes and wood strands as well as types of failure where C, B, and S stands for crushing, bending, and shear failure, respectively.

**Figure 7:**
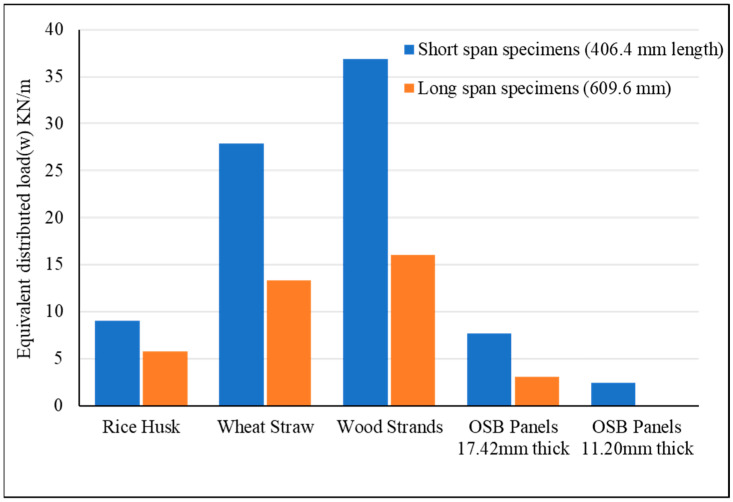
Comparison of equivalent distributed load and bending stiffness for short- and long-span specimens.

The maximum value of normal stress for both sandwich and flat panels tested over short and long spans is shown in Figure 6. This graph shows that sandwich panels with wood strand corrugated cores exhibited greater strength compared to flat OSB panels, as these panels failed under high tensile stress in the bottom layer. Based on their failure modes, the maximum normal stress reported in Figure 6 can be considered the modulus of rupture (MOR) for these panels. However, sandwich panels with wheat straw and rice husk corrugated cores mainly failed due to shear and compressive stresses. The failure modes of all the specimens are reported in Figure 6. Given these failure modes, the maximum tensile stress shown in Figure 6 cannot be regarded as the modulus of rupture (MOR) for sandwich panels with rice husk and wheat straw corrugated cores. As a result, no conclusion can be made about the strength of these sandwich panels compared to flat OSB and wood strand corrugated core sandwich panels. Therefore, to assess the load-carrying capacity of these panels, the equivalent distributed load was calculated and reported in Figure 7.

The distributed load equivalent to the maximum bending load applied at the time of failure was calculated using Equation (2) and is presented in Figure 7. As shown, the load-carrying capacity of all sandwich panels exceeds that of flat OSB panels. The maximum equivalent distributed load for sandwich panels with wood strand corrugated cores was 378% higher for short spans and exceeded by 421% for long spans compared to 17.42 mm (0.75-inch)-thick OSB panels. Wheat straw corrugated core sandwich panels exhibited remarkable performance, with equivalent distributed loads that surpassed the 17.42-mm (0.75-inch)-thick OSB panels by 262% for short spans and 334% for long spans. The thin-walled structure of the corrugated geometry, along with the greater depth of the associated sandwich panels, reduced the normal and shear stresses generated in these panels compared to flat panels under similar bending loads. This reduction in stress contributed to an improved load-carrying capacity for the sandwich panels. Sandwich panels with wood strand corrugated cores exhibited the highest load-carrying capacity, while panels with rice husk cores showed the lowest, and those with wheat straw corrugated cores fell in between. These differences in load-carrying capacity can be attributed to fiber length. Longer fibers provide better overlap, enhance load transfer, and result in a higher load-bearing capacity. In contrast, shorter fibers are less effective in transmitting stresses across the panel, leading to lower mechanical performance. The higher load-carrying capacity of specimens with shorter spans compared to those with longer spans is attributed to the reduced bending moment, lower deflection, and decreased bending deformation.

**Figure 8:**
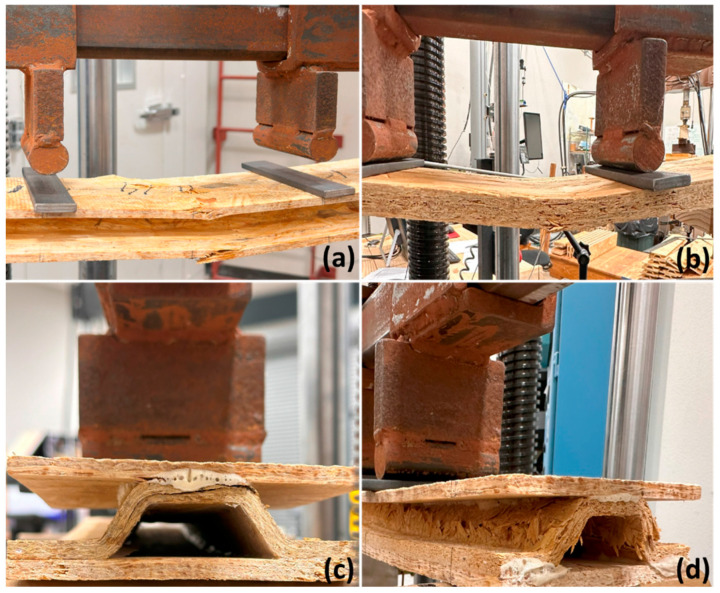
Failure modes for 17.42 mm (0.75-inch)-thick OSB and corrugated core sandwich panels: (**a**,**b**) Simple bending failure for wood strand corrugated sandwich panels and 17.42-mm-(0.75-inch) thick OSB. (**c**) Wheat straw corrugated core panel with delamination within corrugated core. (**d**) Rice husk corrugated core panel with crushing failure along the core.

The failure modes of the specimens given in Figure 6 revealed valuable insights into their structural performance and weaknesses. Both the short-span and long-span flat OSB panels, as well as the wood strand corrugated core sandwich panels, failed primarily due to tensile stress in the bottom layer, as illustrated in Figure 8a,b. This failure mode confirms the high shear and compression strength of these panels, particularly in short-span specimens where shear deformation is significant. Similarly, long-span wheat straw corrugated core sandwich panels also experienced failure due to tension, while some of the short-span specimens failed as a result of either delamination between the core and outer layers or delamination within the corrugated core, as depicted in Figure 8c. This might be due to the waxy and silica-rich surface layer of the wheat straw, which hinders effective bonding [30,37]. Regardless of the span length, sandwich specimens with rice husk corrugated cores failed due to crushing in the slanted ribs of the corrugated cores, as shown in Figure 8d. The short rice fibers, combined with poor bonding among the rice husks, contributed to this crushing failure, reducing the load-carrying capacity of the rice husk sandwich panels. According to Pradhan et al., silica has an adverse effect on the bonding and strength properties of rice husk panels [32]. Nevertheless, it is important to note that the rice husk sandwich panels demonstrated greater bending stiffness and load-carrying capacity compared to the 17.42 mm (0.75-inch)-thick flat OSB panels, as shown in Figure 5 and Figure 7.

**Figure 9:**
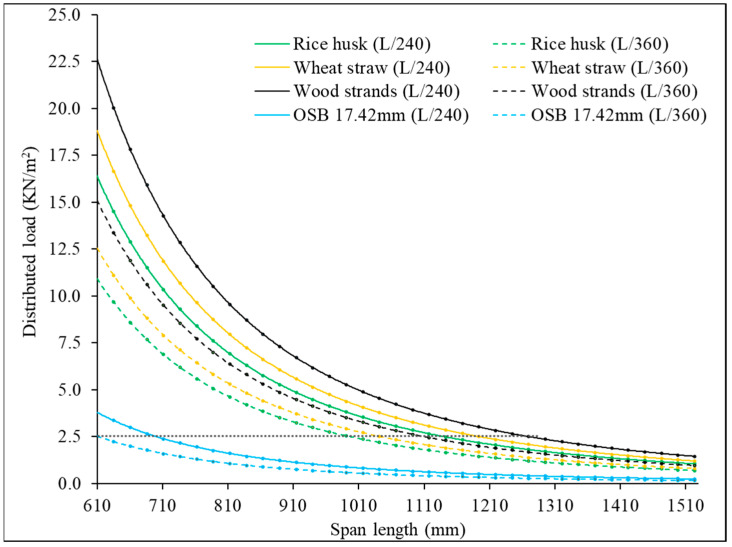
Distributed load for different span lengths under specific deflection limits of L/240 and L/360.

According to the International Building Code (IBC), the allowable deflection for flooring systems is constrained by two limits: L/240 for a combination of dead and live loads and L/360 for live loads only, where L represents the span length [38]. The corresponding length and span lengths for these deflection limits were calculated using Equation (3) and compared to a 17.42 mm (0.75-inch)-thick OSB panel, as illustrated in Figure 9. The analysis shows that corrugated core sandwich panels, particularly those with wood strand corrugated cores, outperform the OSB panels by allowing significantly longer spans, while adhering to the same deflection limits. The IBC’s Section 1607.22 specifies a standard live load of 1.9 kN/m^2^ (40 psf) for residential buildings. Testing of 17.42 mm OSB panels at a span of 609.6 mm (24 inches) yielded a minimum live load capacity of 2.5 kN/m^2^ (53 psf), showing with a dashed line in Figure 9, meeting the IBC requirement for residential use under an L/360 deflection criterion. By comparison, corrugated panels made from rice husk, wheat straw, and wood strands were able to support spans of approximately 980 mm (38.6 inches), 1040 mm (41 inches), and 1100 mm (43.3 inches), respectively, under the same bending load of 2.5 kN/m^2^ (53 psf), while still satisfying the deflection limit of L/360.

For a span length of 609.6 mm (24 inches), these sandwich panels supported significantly higher bending loads than 2.5 kN/m^2^ (53 psf) supported by flat OSB panels: 10.9 kN/m^2^ (228 psf) for rice husk, 12.6 kN/m^2^ (262 psf) for wheat straw, and 15.1 kN/m^2^ (315 psf) for wood strand corrugated cores. This improved load-bearing capacity highlights their potential as viable subflooring materials suitable for high-load applications, such as libraries, manufacturing spaces, and recreational facilities, where typical live load requirements range from 150 psf to 250 psf. These findings suggest that corrugated core sandwich panels made from underutilized materials from agriculture and forestry systems could serve as a viable alternative to traditional OSB panels in structural applications. Their ability to support extended spans, while reducing material consumption and minimizing the need for additional I-joists, offers both economic and functional advantages for sustainable construction.

## 4. Conclusions

This study evaluated the mechanical performance of corrugated sandwich panels made from agricultural residues (rice husk and wheat straw) and wood strands, with comparisons to conventional OSB panels. Flat OSB panels with 11.2 mm (0.5 in) and 17.42 mm (~0.75 in) thicknesses were employed, and two different span lengths of 41 cm (16 inches) and 61 cm (24 inches) were considered. The results from the four-point bending tests revealed notable differences in bending stiffness, equivalent distributed load, and failure modes among the materials. The findings underscore the potential of these biobased materials for structural applications, particularly for flooring applications. Based on the analysis, the following key points were derived:

1. Both rice husk and wheat straw corrugated core sandwich panels demonstrated superior bending stiffness compared to 17.42 mm (0.75-inch)-thick flat OSB panels. Wheat straw panels showed higher bending stiffness of 132% for short spans and 396% for long spans, while rice husk panels achieved 71% and 331% greater for short span and long span, respectively, as that of OSB panels.
2. In terms of equivalent distributed load, which is essential for material selection in building design, wheat straw corrugated core sandwich panels outperformed 17.42 mm (0.75-inch)-thick flat OSB panels by 262% in short spans and 334% in long spans. Rice husk corrugated core sandwich panels also exceeded OSB performance, with 18% more load capacity in short spans and 88% in long spans.
3. Wood strand corrugated core sandwich panels outperformed both rice husk and wheat straw corrugated core sandwich panels and also 17.42 mm (0.75-inch)-thick flat OSB panels. Specifically, these panels were 168% and 496% stiffer than flat OSB panels for short and long spans, respectively. In terms of equivalent distributed loads, they supported 378% and 421% more than flat OSB panels for short and long spans. This exceptional performance is largely due to the geometric structure and longer fiber length of wood strands.
4. All the corrugated panels made from crop residues (rice husk and wheat straw) and wood strands demonstrated superior compliance with International Building Code (IBC) deflection limits (L/240 and L/360) when compared to 17.42 mm (0.75-inch) OSB panels. Under a distributed load of 2.5 kN/m^2^ (53 psf) and satisfying the deflection limit of L/360, sandwich panels with corrugated cores made from rice husk, wheat straw, and wood strands supported spans of approximately 980 mm (38.6 inches), 1040 mm (41 inches), and 1100 mm (43.3 inches), respectively, while 17.42 mm (0.75-inch)-thick flat OSB panels could only span 609.6 mm (24 inches).
5. This performance underscores the potential of underutilized agricultural materials, such as wheat straw and rice husk, and forestry systems, like small-diameter trees processed into wood strands, as a practical biobased option for structural panels. These materials offer practical solutions for achieving the spans and loads required in many flooring applications.

These findings suggest that sandwich panels with corrugated cores fabricated from wheat straw, rice husk, and wood strands offer promising alternatives to conventional flat OSB panels for specific structural applications. The engineered corrugated geometry enhanced the structural performance compared to flat OSB panels, achieving this improvement without a substantial increase in raw materials consumption. Manufacturing these corrugated panels involves hot-pressing the corrugated panels and flat outer layers separately, followed by bonding them together. Therefore, additional research is necessary to evaluate economic feasibility, environmental impact, and additional mechanical properties for successfully introducing these high-performance sandwich panels to the construction market.

## Figures and Tables

**Table 1:** Final dimensions of sandwich panels.

Types of Composite Panels		Average Length (mm)	Average Width (mm)	Average Thickness (mm)	Average Weight (gm)	Average Density (kg/m^3^)
Rice Husk	Short	482.60	125.41	38.27	1044.29	450.90
Wheat Straw		482.60	127.00	38.48	1064.08	451.20
Wood Strands		482.60	127.00	38.65	1146.90	484.09
OSB Panels 0.5″		480.85	127.00	11.20	438.92	641.61
OSB Panels 0.75″		483.13	127.00	17.42	705.29	659.95
Rice Husk	Long	687.50	127.23	38.69	1510.63	446.36
Wheat Straw		686.86	127.00	38.90	1510.00	445.00
Wood Strands		687.78	127.00	38.87	1640.02	483.05
OSB Panels 0.75″		685.80	127.00	17.49	945.51	620.67

**Table 2:** Actual density and thickness of corrugated panels.

Materials	Target Wall Thickness	Actual Thickness (mm)	Target Density	Actual Density (kg/m^3^)
Wheat Straw	6.35	6.84	720	744.79
Rice Husk	6.35	6.97	720	719.62
Wood Strands	6.35	6.99	720	899.86

## Data Availability

The raw data supporting the conclusions of this article will be made available by the authors on request.
